# Greater efficacy of chemotherapy plus bevacizumab compared to chemo- and targeted therapy alone on non-small cell lung cancer patients with brain metastasis

**DOI:** 10.18632/oncotarget.6184

**Published:** 2015-10-20

**Authors:** Ning Tang, Jun Guo, Qianqian Zhang, Yali Wang, Zhehai Wang

**Affiliations:** ^1^ School of Medicine and Life Sciences, University of Jinan-Shandong Academy of Medical Sciences, Jinan, Shandong, China; ^2^ Department of Shandong Cancer Hospital and Institute, Jinan, Shandong, China

**Keywords:** non-small cell lung cancer, brain metastases, chemotherapy, tyrosine kinase inhibitors, epidermal growth factor receptor

## Abstract

Control of non-small-cell lung cancer (NSCLC) with brain metastasis is clinically challenging. This study retrospectively evaluated the efficacy of different adjuvant therapies for 776 cases of advanced NSCLCs with brain metastasis who treated with chemotherapy, chemotherapy plus bevacizumab, tyrosine kinase inhibitor (TKI) alone, or supportive care. The median progression-free survival (mPFS) and median overall survival (mOS) of patients treated with chemotherapy plus bevacizumab were 8.5 and 10.5 months, respectively, which were better than those of patients treated with other three therapies(*P* < 0.01). For patients with EGFR-mutated NSCLC, the efficacy of TKI treatment was not statistically better than that of chemotherapy plus bevacizumab but was significantly better than that of other therapies. Moreover, for patients with EGFR wild-type NSCLC, the mPFS and mOS after chemotherapy plus bevacizumab were greater than those with other two therapies (*P* < 0.01). The local response rate (RR)and disease control rate (DCR)with regimen including pemetrexed were greater than those with regimen including paclitaxel (*P* < 0.05). Chemotherapy plus bevacizumab was more effective for NSCLC patients with brain metastasis. Further studies will investigate the benefit of TKI alone for patients with EGFR-mutated. For patients with EGFR wild-type, chemotherapy plus bevacizumab did improve PFS and OS. Furthermore, regimens including pemetrexed led to a greater RR.

## INTRODUCTION

Lung cancer is the leading cause of cancer-related deaths in the world [1] and non-small cell lung cancer (NSCLC) accounts for approximately 80% of all lung cancer cases [1]. NSCLC is usually diagnosed at the advanced stages of disease, and brain metastasis is a common complication in NSCLC patients, with more than 10% of NSCLC patients presenting with brain metastases at their first hospital visit [2, 3] and 30%–40% of NSCLC patients developing brain metastasis during the course of the disease [4]. The standard treatment protocol for patients with multiple metastases is whole brain radiotherapy (WBRT) [5] and steriotactic radiosurgery (SRS) used to treat solitary or oligo-metastatic disease that contains the following techniques: gamma knife, three-dimensional conformal radiation therapy (3DCRT), and/or intensity modulation radiated therapy (IMRT) [6–9]. For patients with multiple cerebral lesions, the prognosis is still poor; the median overall survival (mOS) was reported to be only 4–6 months after radiotherapy [10, 11] or only approximately 1 month without treatment. Furthermore, the quality of life of these patients is also very poor [5]. Thus, more effective treatment regimens or strategies to control NSCLC with brain metastasis are urgently needed.

To date, the best optimal chemotherapy regimens for NSCLC patients with brain metastases are still deba Table and sometimes are controversial, although platinum compounds are still basically the first-line NSCLC chemotherapy in the clinic. Recent studies showed the effectiveness of pemetrexed treatment in NSCLC patients with brain metastasis [12–14], and molecular targeted drugs have offered new hope to these patients [15]. Bevacizumab, the most widely used drug in anti-angiogenic therapy, has also been shown to improve response rates, progression-free survival (*PF*S), and OS compared to chemotherapy alone [16–18]. However, with the fear of tumor-related intracranial hemorrhage (ICH) [19], the use of bevacizumab to treat NSCLC patients has remained fairly rare, except in one study reported by Socinskiet al. [15] who suggested that addition of bevacizumab to various chemotherapy regimens or erlotinib for treating NSCLC patients with brain metastases is safe. However, the efficacy of this strategy remains to be confirmed. For NSCLC patients, targeted therapy is progressing rapidly [20–30]. One example is the targeting of mutations of epidermal growth factor receptor (EGFR). Several randomized, open-label, phase III clinical trials have compared tyrosine kinase inhibitors (TKIs) with the routine chemotherapy, and the data suggest that the response rate (RR) and PFS of patients with EGFR-mutated NSCLC who receive EGFR-TKIs as a first-line treatment are significantly greater than those of patients who receive chemotherapy, although the OS did not differ significantly between these two treatments [20–30]. In addition, NSCLC patients with brain metastasis were excluded from these studies [20–30]. Patients with EGFR-mutated NSCLC do not usually receive TKI treatment in China due to economic or other reasons. Thus, in this study, we compared the effectiveness of different treatment regimens in NSCLC patients with brain metastasis using a retrospective setting to provide valuable information for future clinical treatment of NSCLC patients with brain metastasis.

## RESULTS

### Patient characteristics

For the 776 patients included in this study, the median age was 58 years;423 were male and 353 female. All patients had brain metastasis and 37% also had pulmonary metastasis, 24% had ossary metastasis, and 7% had hepatic metastasis. These patients were treated with chemotherapy alone, chemotherapy plus bevacizumab, TKIs alone, or supportive care. However, all patients, except 61 who received only supportive care, had received concurrent radiotherapy (WBRT or SRS). The characteristics of these patients are shown in Table 1, and no significant differences were observed between the four treatment groups.

**Table 1:** Characteristics of patients and treatment selections [n(%)]

Characteristics (%)	Overall (*N* = 776)	Chemotherapy alone (*N* = 523)	Chemotherapy + bevacizumab (*N* = 117)	TKIs alone (*N* = 75)	Supportive care(*N* = 61)	*P*
Age (years)						
> 50	546(70.4)	376(71.9)	65(55.6)	53(70.7)	52(85.2)	
≥ 50	230(29.6)	147(28.1)	52(44.4)	22(29.3)	9(14.8)	> 0.05
Sex						
male	423(54.5)	292(55.9)	53(45.3)	38(50.7)	40(65.6)	
female	353(45.5)	231(44.2)	64(54.7)	37(49.3)	21(34.4)	> 0.05
ECOG						
0–2	719(92.7)	498(95.2)	112(95.7)	69(92.0)	40(65.6)	
≥ 3	57(7.3)	25(4.8)	5(4.3)	6(8.0)	21(34.4)	> 0.05
Histologic type						
AC	726(93.6)	484(92.5)	110(94.0)	74(98.7)	58(95.1)	
SCC	50(6.4)	39(7.5)	7(6.0)	1(1.3)	3(4.9)	> 0.05
EGFR						
mutation	416(53.6)	249(47.6)	70(59.8)	75(100)	22(36.1)	
wild-type	360(46.4)	274(52.4)	47(40.2)	0	39(63.9)	
Other sites of metastases						
lung	289(37.2)	190(36.3)	47(40.2)	38(50.7)	14(23.0)	
bone	188(24.2)	117(22.4)	35(29.9)	26(34.7)	10(16.4)	
liver	53(6.8)	36(6.8)	6(5.1)	6(8.0)	5(8.2)	
adrenal gland	28(3.6)	20(3.8)	3(2.6)	2(2.7)	3(4.9)	
pleura	25(3.2)	16(3.1)	7(6.0)	0	2(3.3)	
kidney	20(2.6)	11(2.1)	3(2.6)	3(4.0)	3(4.9)	
Single lesion of the CNS						
Yes	203(26.2)	144(27.5)	30(25.6)	23(30.7)	6(9.8)	
No	573(73.8)	379(72.5)	87(74.4)	52(69.3)	55(90.2)	> 0.05
Chemotherapeutic regimen						
Taxol	344(44.3)	286(54.7)	58(49.6)			
Pemetrexed	278(35.8)	220(42.1)	58(49.6)			
Gemcitabine	16(2.1)	15(2.9)	1(0.1)			
Other	2(0.3)	2(0.4)	0			
Response						
CR+PR	228(29.4)	156(29.8)	46(39.3)	26(34.7)	0	
SD	266(34.3)	184(35.2)	42(35.9)	26(34.7)	14(23.0)	
DCR	494(63.7)	340(65.0)	88(75.2)	52(69.3)	14(23.0)	
PD	282(36.3)	183(35.0)	29(24.8)	23(30.7)	47(77.0)	

Abbreviations: EGFR, epidermal growth factor receptor; ECOG, Eastern Cooperative Oncology Group; CR, complete response; PR, partial response; SD, stable disease; DCR, disease control rate; PD, progression of disease; TKIs, tyrosine kinase inhibitors; AC, adenocarcinoma; SCC, squamous cell carcinoma

### Association of treatment selections with patient survival

**Figure 1:**
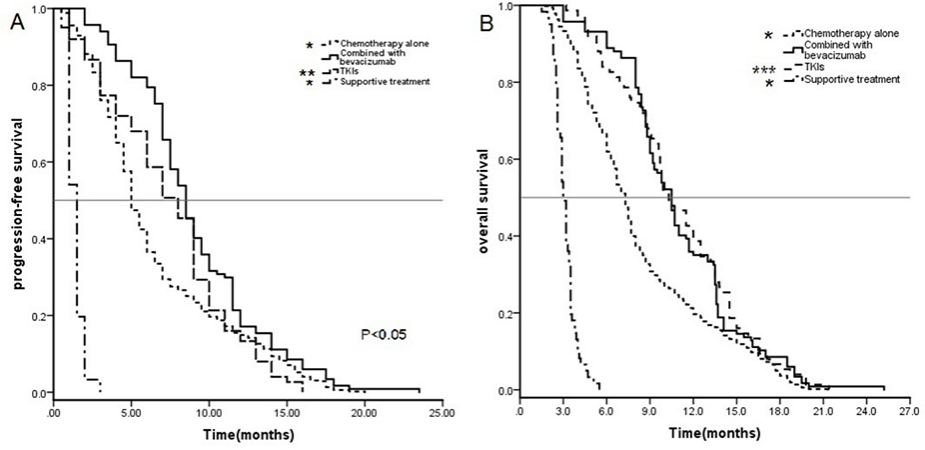
Kaplan–Meier curves for progression-free survival (PFS) (A) and overall survival (OS) (B) of all 776 patients *P < 0.01for chemotherapy plus bevacizumab compared to chemotherapy alone; **P < 0.05 for chemotherapy plus bevacizumab compared to TKIs alone; ***P > 0.05 for chemotherapy plus bevacizumab compared to supportive care.

PFS and OS data stratified by the different treatments were analyzed using Kaplan–Meier curves and the log-rank test (Figure 1). Specifically, the mPFS of all 776 patients was 5.5 months (95% confidence interval [CI]:5.1–5.8 months), and the mPFS times after chemotherapy alone, chemotherapy plus bevacizumab, TKIs alone, and supportive care were 5.0 months (95% CI: 4.7–5.2 months), 8.5 months (95% CI:7.7–9.3 months), 8.0 months (95% CI:6.8–9.1 months), and 1.5 months (95% CI:1.3–1.6 months), respectively. The mPFS after chemotherapy plus bevacizumab was significantly greater than those of the other three treatment groups (*P* < 0.05), including even the TKI treatment group (*P* = 0.024).

The mOS of all 776 patients was 7.7 months (95% CI:7.4–7.9 months), and the mOS times after chemotherapy alone, chemotherapy plus bevacizumab, TKIs alone, and supportive care were 7.3 (95% CI:6.9–7.6), 10.5 (95% CI:9.7–11.3), 10.3 (95% CI:9.0–11.5), and 3.0 months (95% CI:2.8–3.2 months), respectively. The mOS after chemotherapy plus bevacizumab was significantly greater than that after chemotherapy alone and after supportive care (*P* < 0.01), but not statistically different from that with the TKI treatment (*P* = 0.836).

### Association of different treatments with survival of patients with EGFR mutated NSCLC

**Figure 2:**
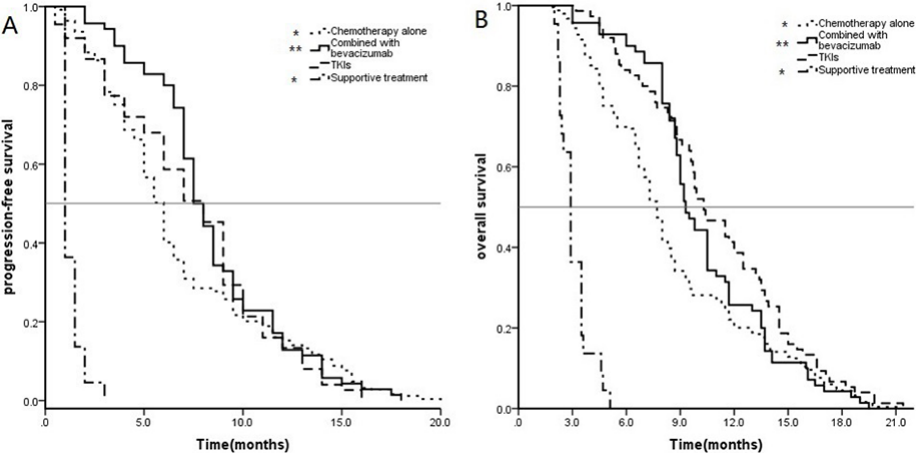
Kaplan–Meier estimates of (A) progression-free survival (PFS) and(B) overall survival (OS) in 416 patients with EGFR mutated NSCLC *P < 0.05 for chemotherapy alone versus TKI treatment alone and **P > 0.05 for chemotherapy plus bevacizumab versus TKI treatment alone.

PFS and OS data for the 416 patients with EGFR mutated NSCLC were stratified by the different treatments for analysis with Kaplan–Meier curves and the log-rank test (Figure 2). Specifically, the mPFS of these 416 patients was 6.5 months (95% CI: 6.1–6.8 months), whereas the mPFS times after chemotherapy alone, chemotherapy plus bevacizumab, TKIs alone, and supportive care were 6.0 (95% CI: 5.6–6.3), 7.5 (95% CI:6.8–8.2), 8.0 (95% CI:6.8–9.1), and 1.0 month(s) (95% CI:0.8–1.2), respectively. The mPFS after TKI treatment alone was significantly greater than that after chemotherapy alone and after supportive care (*P* < 0.01), but not statistically different from that after chemotherapy plus bevacizumab (*P* = 0.411).

The mOS of these 416 patients was 8.3 months (95% CI:7.9–8.7), whereas the mOS after chemotherapy alone, chemotherapy plus bevacizumab, TKIs alone, and supportive care was 7.7 (95% CI:7.3–8.0), 9.3 (95% CI: 8.5–10.1), 10.3 (95% CI:9.0–11.5), and 2.9 months (95% CI:2.6–3.1 months), respectively. The mOS after TKI treatment alone was significantly greater than that after chemotherapy alone and after supportive care (*P* < 0.01), but was not statistically different from that after chemotherapy plus bevacizumab (*P* = 0.130).

### Association of different treatments with survival of patients with wild type EGFR NSCLC

**Figure 3:**
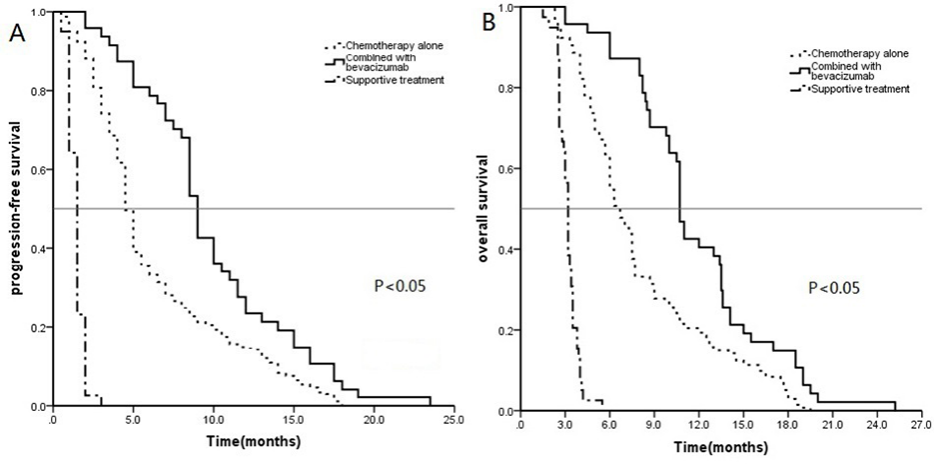
Kaplan–Meier curves for progression-free survival (PFS) (A) and overall survival (OS) (B) in 360 patients with EGFR wildtype NSCLC

The PFS and OS data for the 360 patients with EGFR wild type NSCLC were stratified by the different treatments for analysis with Kaplan–Meier curves and the log-rank test (Figure 3). Specifically, the mPFS of these 360 patients was 4.5 months (95% CI:4.2–4.8 months), whereas the mPFS after chemotherapy alone, chemotherapy plus bevacizumab, and supportive care was 4.5 (95% CI:4.2–4.8), 9.0 (95% CI: 8.4–9.5), and 1.5 months (95% CI:1.3–1.6 months), respectively. The mPFS after chemotherapy plus bevacizumab was significantly greater than that after chemotherapy alone and after supportive care (*P* < 0.01).

The mOS of these 416 patients was 6.3 months (95% CI: 5.7–6.8 months), whereas the mOS after chemotherapy alone, chemotherapy plus bevacizumab, and supportive care group was 6.7 (95% CI: 6.2–7.1), 10.7 (95% CI: 10.3–11.1), and 3.2 months (95% CI: 3.0–3.4 months), respectively. The mOS after chemotherapy plus bevacizumab was significantly greater than that after chemotherapy alone and after supportive care (*P* < 0.01).

### Association between different cytotoxic drugs and survival in patients who received adjuvant chemotherapy

Among the total of 776 patients, 622 patients were treated with adjuvant chemotherapy. We assessed the treatment responses for different cytotoxic drugs as the first-line treatment (Table 2). Among patients who received a pemetrexed regimen (*n* = 278) oral taxane regimen (*n* = 344), the concurrent effect on the overall response among the different cytotoxic drugs did not differ significantly (*P* > 0.05), whereas the RR and DCR for a local response to regimens including pemetrexed or taxane were significantly better than those for a regimen including paclitaxel(*P* < 0.05).

**Table 2:** Overall and local response of NSCLC patients with brain metastasis to different cytotoxic drugs [n (%)]

Response	Regimen including pemetrexed	Regimen including taxane	*P*
Overall			
CR	0	0	
PR	91(32.7)	91(26.5)	0.087
CR + PR	91(32.7)	91(26.5)	
SD	89(32.0)	132(38.3)	0.10
DCR	180(64.7)	223(64.8)	0.33
PD	98(35.3)	111(35.2)	
Local			
CR	6(2.2)	0	
PR	110(39.6)	104(30.2)	0.015
CR + PR	116(41.8)	104(30.2)	0.003
SD	83(29.9)	116(33.7)	0.30
DCR	199(71.6)	220(63.9)	0.044
PD	79(28.4)	124(36.1)	

### Toxicity and feasibility of different therapies

Drug toxicity was evaluated according to WHO criteria, and no grade 3/4 adverse reactions occurred among patients in all four different groups, even among the 57 patients with a performance status ≥ 3 (Table 3). There was no significant differences in drug toxicity among the three treatments not including supportive care group (*P* > 0.05). Also, no cases of ICH, hypertension, epistaxis, protenuria, or hemoptysis occurred after any treatment (Table 3).

**Table 3:** Response rates and Adverse effects of each treatment [n (%)]

	Chemotherapy alone	Chemotherapy + bevacizumab	TKIs alone	Supportive care
**Response**				
CR+PR	156(29.8)	46(39.3)	26(34.7)	0
SD	184(35.2)	42(35.9)	26(34.7)	14(23.0)
DCR	340(65.0)	88(75.2)	52(69.3)	14(23.0)
PD	183(35.0)	29(24.8)	23(30.7)	47(77.0)
**Adverse reaction Grade 2**				
Hematological				
Neutropenia	210(40.2)	40(34.2)	26(34.7)	2(3.3)
Anemia	62(11.9)	13(11.1)	8(10.7)	2(3.3)
Thrombocytopenia	125(23.9)	26(22.2)	7(9.3)	1(1.6)
Non-hematological				
Asthenia	283(54.1)	53(45.3)	30(40.0)	5(8.2)
Anorexia	260(49.7)	54(46.2)	32(42.7)	0
Vomiting	30(5.7)	6(5.1)	4(5.3)	0
Diarrhea	27(5.2)	5(4.3)	4(5.3)	1(1.6)
Constipation	190(36.3)	40(34.2)	25(33.3)	0
Rash	29(5.5)	7(6.0)	6(8.0)	0
Weight loss ≥ 3kg	39(7.5)	5(4.3)	3(4.0)	3(4.9)

Abbreviations: CR, complete response; PR, partial response; SD, stable disease; DCR, disease control rate; PD, progression of disease; TKIs, tyrosine kinase inhibitors.

## DISCUSSION

In the current study, we assessed the effectiveness of different therapeutic regimens on PFS and OS of 776 NSCLC patients with brain metastasis. We found that chemotherapy plus bevacizumab resulted in an mPFS of 8.5 months and an mOS of 10.5 months and that these PFS and OS were significantly greater than those with chemotherapy alone or supportive care. However, the efficacy of the maintenance TKI treatment of patients with EGFR-mutated NSCLC did not differ significantly from that of chemotherapy plus bevacizumab, but was significantly better than that of chemotherapy alone or supportive care. Moreover, the mPFS and mOS after chemotherapy plus bevacizumab in patients with EGFR wild-type NSCLC were significantly better than those after chemotherapy alone or supportive care. Our current study demonstrated that chemotherapy plus bevacizumab was more effective on NSCLC patients with brain metastasis and the adverse reactions were tolerable. Further prospective studies are needed to confirm our current findings.

Tumor metastasis is a multiple mechanistic process by which tumor cells from a primary site invade and metastasize to a secondary site, while metastasis to the brain is even more complex. This is because tumor brain metastases have their own characteristics. Tumor metastasis to the brain can be divided into six basic steps: escape from the primary site (escape), spreading into the circulation system (dissemination), adhesion and vascular wall (attachment), penetration of the blood–brain barrier into the brain parenchyma (extravasation), brain micro-environment interactions (interaction), and growth in the brain (or secondary site) (survival and proliferation) [31]. Kienast et al. [32] established a mouse brain metastasis model by injecting tumor cells into the carotid artery and used multiphoton laser scanning microscopy to image the single steps of metastasis formation in real time. Their findings revealed that the most critical step in tumor metastasis to the brain is the blockage of tumor cells in the microvessels, which allows them to effectively penetrate the vessel walls, closely adhere to perivascular cells, and form micrometastasis in the brain [32]. If tumor cells fail to undergo these four steps, they become motionless, decline, or die. Overall, only 1.0% ∼2.4% of lung cancer cells injected into the carotid artery completed the metastasis process, which is lower than the 4.7% ∼7.0% of melanoma cells that complete the metastasis process [32]. These results demonstrated that tumor cells exhibit different biological behaviors in these fourth key steps, i.e., the support of melanoma growth was a vascular co-option that was dependent on the existing blood vessels, and angiogenesis supported adenocarcinoma metastatic cell growth. Thus, these previous findings provided a mechanistic basis for anti-angiogenesis therapy for lung cancer metastasis to the brain.

Bevacizumab is a recombinant, humanized monoclonal antibody that directly targets vascular endothelial growth factor (VEGF) [16]. The latter regulates tumor-associated angiogenesis. Bevacizumab, combined with platinum-based doublet regimen, is a US Food and Drug Administration-approved first-line treatment for patients with unresectable, locally advanced, recurrent, or metastatic NSCLC [16, 33, 34]. In a previous phase III study reported by the Eastern Cooperative Oncology Group (ECOG), bevacizumab accession can significantly improve the OS and PFS of NSCLC patients compared to carboplatin/paclitaxel alone [16]. The results of our current study showed that chemotherapy plus bevacizumab as the first-line treatment regimen was much more effective than chemotherapy alone and comparable with TKI treatment alone. With respect to the adverse effects of this regimen, there were no grade 3–4 adverse reactions observed in patients and no adverse reactions related to bevacizumab, such as ICH, hypertension, epistaxis, proteinuria, and hemoptysis, occurred. Thus, our finding suggests that chemotherapy plus bevacizumab as the first-line treatment had a better curative rate and tolerable adverse reactions in NSCLC patients with brain metastasis, especially in patients with EGFR wild type NSCLC.

Furthermore, previous studies have considered that the effectiveness of chemotherapy is subject to a presumed lack of effectiveness due to the blood–brain barrier [12]. Actually two reasons were behind the poor response: i) Utilization rate of drug at the site of action remains low, and ii) Intrinsic or acquired resistance to anticancer agents [35]. This may be because overexpression or mutation of drug-targeting enzymes leads to natural or acquired resistance [35]. Pemetrexed has been therefore developed to solve these problems by inhibiting at least three types of enzymes [36]. Simultaneous inhibition of these three enzymes at multiple sites could lead to improvement of drug effectiveness. Thus, use of pemetrexed could overcome intrinsic or acquired drug resistance, and previous clinical studies confirmed the broad usefulness of pemetrexed in the treatment of a variety of solid tumors [36–38]. These enzymes include dihydrofolate reductase (DHFR), thymidylate synthetase, glycinamide-ribonucleotide-formyl transferase [36–39]. In our current study, the RRs and DCRs for different cytotoxic drugs did not differ significantly, whereas the local RR and DCR for regimens including pemetrexed were significantly greater than those for regimens including paclitaxel. Our study included five patients with single brain lesions that showed CR after the treatment. This implies that pemetrexed was able to pass through the blood–brain barrier to reach tumor lesion. Thus, a regimen including pemetrexed may outperform the other cytotoxic drugs.

In addition, a previous phase II clinical trial showed that WBRT combined with the molecular targeted drug erlotinib improved the OS of NSCLC patients with brain metastasis, especially those with EGFR mutated NSCLC [39]. Multiple studies suggest that WBRT combined with TKIs was able to improve the control of disease progression and was well tolerated [40]. The efficiency of WBRT plus TKIs was about 70% in patients independent of EGFR status [40], which was significantly higher than that of WBRT or chemotherapy alone. Kim et al. treated 23 patients with EGFR-TKIs as the first line and showed an mPFS of 7.1 months and an mOS of 18.8 months, leading to a 82.6% control rate [41]. However, the use of EGFR-TKIs as a first-line treatment remains controversial [41]. Moket al. analyzed the relevant literature and argued that OS may not differ between EGFR-TKIs as the first-line or second-line therapy, but that this treatment strategy can prolong the mPFS and delay the time of radiotherapy as a first-line treatment to improve patients' quality of life [42]. In the current study, the efficacy of TKI maintenance treatment in patients did not differ significantly from that of chemotherapy plus bevacizumab, even in patients with EGFR mutation. However, further studies are needed before a conclusion can be drawn, because our current study included only 75 patients who received this treatment.

## MATERIALS AND METHODS

### Patients

A total of 794 NSCLC patients with brain metastasis were treated at Shandong Cancer Hospital & Institute between January 2013 and January 2015. Eighteen patients were excluded because: i) The pathological type of tumor was not confirmed in 7 patients; ii) EGFR mutation status was unknown in 3 patients, and iii) Survival data were not available in 8 patients. Thus, 776 NSCLC patients with brain metastasis were included in this retrospective analysis. Among them, 50 patients were lung squamous cell carcinoma and 726 were adenocarcinoma. There were 416 NSCLC patients with EGFR mutation, whereas 360 patients had wild-type EGFR. In these 416 EGFR-mutated NSCLC cases, 232 had Exon 19 deletion mutation, whereas 184 had Exon 21:L858R or L861Q mutations. The treatment regimens included adjuvant chemotherapy or chemotherapy plus the targeted therapy (Table 1). In addition, all 776 patients, except 61 patients who only received supportive care, underwent concurrent radiotherapy (WBRT or SRS). Among the 416 patients with EGFR-mutated NSCLC, 249 were treated with chemotherapy alone, 22 with supportive care, 75 with TKIs alone, and 70 with adjuvant chemotherapy plus bevacizumab treatment, including a pemetrexed regimen (*n* = 37) or a taxane regimen (*n* = 33). All the first-line chemotherapy were basis of platinum compounds, and pemetrexed only for these patients. There were also 93 cases with PS ≥ 3 received different regimens of treatment and these patients had a strong demand for more treatment; thus, we also included them in this retrospective analysis. Among the 360 patients with wild-type EGFR, 274 were treated with chemotherapy alone, 39 with supportive care, and 47 with adjuvant chemotherapy plus bevacizumab treatment. This study was approved by the Ethics Committee of Shandong Cancer Hospital & Institute (Shandong, China). The patients or their guardians signed an inform consent form before participation in this study.

### Data collection and evaluation criteria

We collected data regarding all clinicopathological characteristics, treatment responses, and survival from patients' medical records. The treatment responses were evaluated based on the RECIST 1.1 guidelines and classified as complete response (CR), partial response (*P*R), stable disease (SD), and progression of disease (*P*D). CR and PR were included in the RR, whereas CR, PR, and SD were included in the disease control rate (DCR). Moreover, all 776 patients were followed up for a median duration of 11.2 months, and the last follow-up date was in May 2015. Survival data were collected through an active follow-up based on the verification of the vital status of these patients. OS was defined as the time from the date patients received the first-line chemotherapy to death or last follow-up, whereas PFS was defined as the time from the date patients received the first-line chemotherapy to disease progression or death. During the follow-up period, 773 patients developed distant metastasis or local recurrence or died of the disease.

### Statistics analysis

All statistical analyses were performed using the Statistical Package for the Social Sciences version 17.0 software (SPSS Inc., Chicago, IL, USA). RRs among these patients were compared and analyzed using the χ^2^ test, and Fisher's exact test was performed to analyze categorical variables. The Cox regression model was used to identify independent prognostic factors for NSCLC. mPFS and mOS were calculated using the Kaplan–Meier curves and statistically analyzed using the log-rank test. Two-sided *p* values ≤ 0.05 were considered statistically significant.
